# A Case of COVID-Associated Nephropathy (COVAN)

**DOI:** 10.7759/cureus.30872

**Published:** 2022-10-30

**Authors:** Taylor Craig, Moiz Ansari, Parker Foster, Yasir Abdelgadir, Amro Abdelghani, Pinky Jha

**Affiliations:** 1 Internal Medicine, Medical College of Wisconsin, Milwaukee, USA; 2 Internal Medicine, Medical College of Wisconsin, Wauwatosa, USA

**Keywords:** collapsing glomerulopathy, hiv associated nephropathy (hivan), apolipoprotein l1, covan, covid

## Abstract

Collapsing glomerulopathy is a variant of focal segmental glomerulosclerosis (FSGS) causing rapid renal failure. There has been an emergence of these cases among African American patients with COVID-19, especially those with the apolipoprotein L1 (APOL1) allele. We present a case of an African American patient with COVID-19 who tested positive for the APOL1 allele in the setting of acute renal deterioration. This provides a partial explanation for the increased burden of kidney failure in this population. As cases of COVID-19 persist, COVID-associated nephropathy (COVAN) should be suspected in patients with acute kidney injury and treatment tailored accordingly.

## Introduction

Collapsing glomerulopathy, a variant of focal segmental glomerulosclerosis (FSGS), is characterized by rapidly progressive renal failure in the setting of the segmental and widespread collapse of the glomerular capillaries marked by significant hypercellularity of podocyte [1]. There has been an emergence of these cases among African American patients with COVID-19, especially those with the apolipoprotein L1 (APOL1) allele [2]. This new entity is termed COVID-associated nephropathy (COVAN) and is becoming increasingly prevalent in areas where the APOL1 allele is endemic [1].

The current first-line treatment for FSGS is high-dose glucocorticoids, with immunosuppressive therapy with calcineurin inhibitors (CNIs) reserved for steroid-resistant/steroid-intolerant patients. Glucocorticoids are associated with a remission rate of approximately 30% compared to about 50% in patients treated with CNI, although outcomes are poorer in the collapsing variant [2]. The consideration of biopsy is a decision that varies by institution. The Columbia classification (Col-class) for FSGS classifies biopsied specimens into five variants based on histopathology, yet its utility in identifying a treatment response among variants or improving outcomes has yet to be proven [3].

## Case presentation

**Figure 1:**
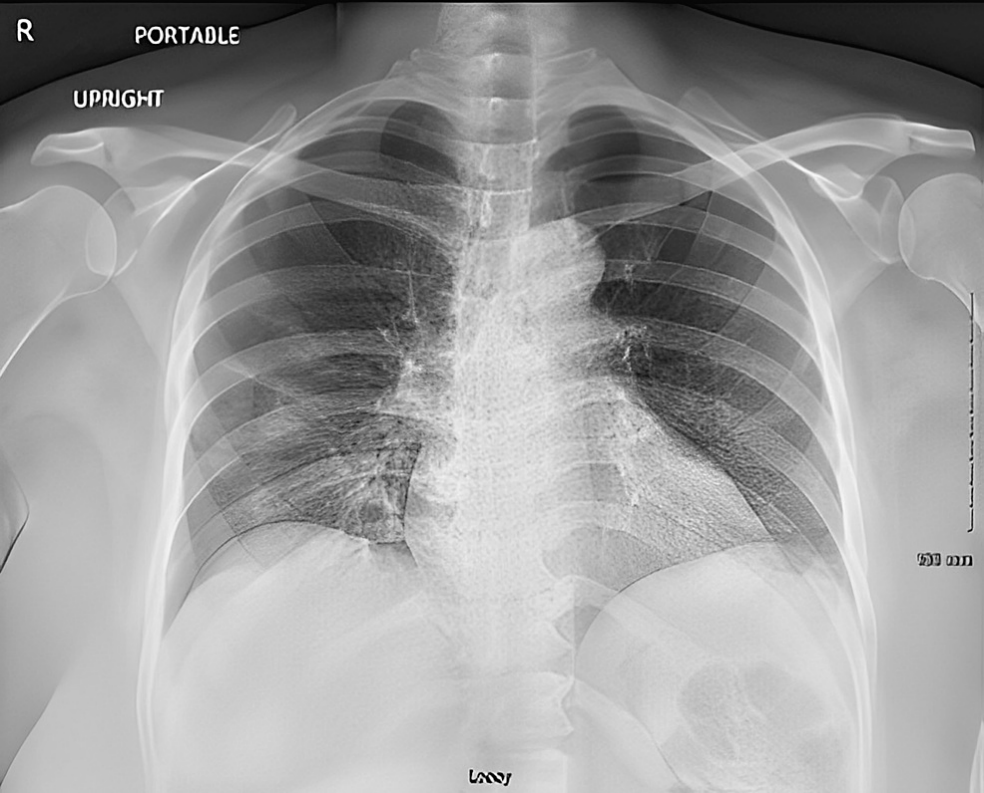
Image of chest x-ray on admission showing interstitial opacities in lung bases.

**Figure 2:**
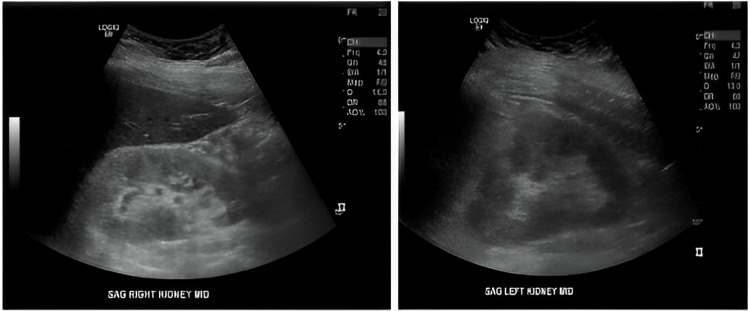
Images of bilateral kidneys showing normal size, echotexture, and parenchymal thickness without hydronephrosis.

Here, we present a 52-year-old African American female with a past medical history of atrial fibrillation, hypertension, and obesity who presented with progressive cough, fever, fatigue, and shortness of breath for one week. She also endorsed loss of sense of smell and taste with loss of appetite and diarrhea during this period. The patient was fully unvaccinated and, upon arrival at the emergency department, tested positive for COVID-19. Upon admission, the patient was on day seven of symptoms and, without hypoxia, did not qualify for remdesivir or dexamethasone therapies. She also had an acute kidney injury (AKI) with creatinine (Cr) of 2.25 mg/dL and blood urea nitrogen (BUN) of 44 mg/dL, with her baseline Cr at 1.09. Her fractional excretion of sodium (FeNa) was <1%, and urinalysis showed a significant proteinuria of >600 mg/dL, the presence of red blood cell (RBC) casts, along with an elevated urine protein/creatinine ratio of 11.06, and an elevated alpha 2 fraction on electrophoresis of 1.17 g/dL. Further workup included imaging (chest x-ray (Figure 1) and bilateral lower extremity ultrasound) to evaluate her shortness of breath and renal ultrasound (Figure 2) to evaluate her decline in kidney function. Basic metabolic panel (BMP), complete blood count (CBC), C3 and C4, anti-neutrophil cytoplasmic antibody (ANCA), and urine specific gravity were ordered per nephrology recommendations and were unremarkable aside from the aforementioned rise in BUN and creatinine. The infectious disease service was also consulted and ordered blood cultures, a *Staphylococcus aureus* respiratory nucleic acid amplification test (NAAT), stool Shiga-like toxin to evaluate the alternative causes for diarrhea, urine legionella, *Streptococcus pneumoniae*, hepatitis C antibody, hepatitis B surface antigen, and human immunodeficiency virus (HIV) antigen/antibody screen, all of which were unremarkable. The patient initially received 1.5 L of Lactated Ringers’ solution given as a bolus in addition to the maintenance of Lactated Ringers' solution at 100 mL/hr; however, her urine output did not respond to fluid resuscitation and remained at 0.1-0.4 mL/kg/hr until day four of hospitalization when the fluids were discontinued due to lack of benefit. Given her rapid rate of rise in creatinine, nephrotic-range proteinuria, and hematuria in the absence of obstructive pathology on renal ultrasound, an acute glomerular process was suspected. With concern for COVAN, the patient was presented at a nephrology conference and ultimately found to be APOL1+ through serology, consistent with COVAN. Nephrology did not recommend biopsy or steroids given the lack of treatment guidelines for this entity, but because the patient’s respiratory status worsened, she was treated with 10 mg of dexamethasone two times per day and was discharged on 6 mg of dexamethasone daily for four days. Her renal function subsequently improved from a peak of Cr 4.31 to 2.26 at discharge (Table 1).

**Table 1:** Trend in blood urea nitrogen and creatinine throughout hospitalization. IVF: intravenous fluids.

				IVF stopped	Steroids started					
Hospital day	0	1	2	3	4	5	6	7	8	9
Creatinine (mg/dL)	2.25	2.31	2.22	3.73	4.01	4.31	4.30	3.53	2.43	2.26
Blood urea nitrogen (mg/dL)	44	52	57	62	78	83	87	82	68	64

## Discussion

Here, we present a case of a patient with renal injury in the setting of COVID pneumonia who was also found to be positive for the APOL1 allele. Approximately 14% of the African American population is homozygous for the APOL1 G1 or G2 alleles, providing a partial explanation for the increased burden of kidney failure in the African American population [1]. Several other viruses have been associated with collapsed glomerulopathy, notably HIV, CMV, parvovirus, and Epstein-Barr virus (EBV) [4]. The pathology lies in the activation of interferon pathways. The APOL1 risk allele is activated by the interferon-chemokine pathway. Once activated, it induces macrophages to engulf glomerular epithelial cells, disrupts mitochondrial homeostasis, and ultimately leads to glomerular epithelial cell death [1]. AKI is not uncommon in COVID-19 patients, with some studies suggesting that nearly 45% of ICU patients have AKI, many of whom end up requiring kidney replacement therapy and therefore are at an increased risk of mortality [4].

Renal biopsy was not ultimately performed on this patient; therefore, it cannot be definitively concluded that this patient had collapsing FSGS; however, an interesting discussion is posed nonetheless. The patient presented with a rapid rise in creatinine with significant proteinuria and RBC casts in the setting of COVID-19, suggestive of glomerular injury. The patient’s lack of response to intravenous fluids, extensive rule out of other possible differentials, and subsequent improvement in creatinine only after the cessation of fluid resuscitation and start of steroid treatment may be a coincidence; however, given the patient’s positive serum apolipoprotein, we believe COVAN to be a strong differential.

## Conclusions

As cases of COVID-19 persist, COVAN should be suspected as a differential diagnosis in patients with acute kidney injury. The relationship between COVID-19 and collapsing glomerulopathy in the setting of APOL1 gene carriers can have important geographical and public health implications. Given that the use of high-dose steroids and immunosuppressive drugs has yielded mixed results, resources should be directed toward investigating the early use of anti-inflammatory agents in at-risk patients to counter the risk of kidney injury and improve overall mortality.
